# Supplementary material for: Content-rich biological network constructed by mining PubMed abstracts
Source: BMC Bioinformatics. 2004 Oct 8;5:147. doi: 10.1186/1471-2105-5-147 (PMC528731; doi:10.1186/1471-2105-5-147)
Supplement: Additional File 5 — The original Chilibot query results of the term "long-term potentiation (LTP)" and 22 other terms, limiting the latest references analyzed to the years 1990, 1995, 2000, and 2004. [file 1471-2105-5-147-S5.bz2 › chilibotAdditionalFile5/ltp2004/html/PKA_NMDA.html]

 


 **PKA** and **NMDA** 
  
Found 120 abstracts in PubMed,  **30 abstracts were retrieved and analyzed**.  


---

 Search Google  |
 PDF files only 
|  EDU domain only 

---

**Interactive relationship** (e.g. stimulation, inhibition, etc)

- In these neurons, protein kinase A  **PKA**  up regulated the  **NMDA**  evoked calcium i increase.  Ref: 12392174 Vis NeurosciVis Neurosci, 1998
- This enhancement is probably associated with phosphorylation of  **NMDA**  receptors mediated by the simultaneous activation of PKC and  **PKA** .  Ref: 12012161 Exp Brain Res, 2002
- **NMDA**  evoked calcium i increase in salamander retinal ganglion cells modulation by  **PKA**  and adrenergic receptors.  Ref: 12392174 Vis NeurosciVis Neurosci, 2002
- In the presence of 8 bromo cAMP or forskolin to stimulate  **PKA** , the elevation level of calcium i induced by  **NMDA**  became even higher.  Ref: 12392174 Vis NeurosciVis Neurosci, 2002
- Application of the specific activator of cAMP dependent protein kinase A  **PKA** , Sp cAMPS triethylamine salt, increased the amplitude of the  **NMDA**  receptor mediated EPSPs at the rest but not at hyperpolarized potentials.  Ref: 12012161 Exp Brain Res, 2002
- As to subcellular neurochemical mechanisms of sensitization, the activation of three main cascades is indispensable, 1 D1 dopamine receptors  **PKA**  phospho 34Thr DARPP 32 PP 1 cascade activated by psychostimulant induced enhancement of DA release in the accumbens, 2  **NMDA**  receptors and CaM KII activated by enhanced release of glutamate, 3 activation of MAP kinase cascade by BDNF and beta 1 subunit of G protein.  Ref: 12642909 Seishin Shinkeigaku Zasshi, 2002
- Coapplication of the PKC and  **PKA**  activators yielded  **NMDA**  EPSPs similar to those seen in Str neurons of MW rats.  Ref: 12012161 Exp Brain Res, 2002
- The  **NMDA**  receptor 1 subunit NR1 is phosphorylated by protein kinase A  **PKA**  on serine 890 and 897.  Ref: 12435416 Neuroscience, 2002
- Because AMPA receptor  **PKA**  phosphorylation and synaptic localization are regulated by similar  **NMDA**  receptor CaN signaling pathways linked to hippocampal long term depression, this regulation of AKAP79 150 postsynaptic targeting might be important for synaptic plasticity.  Ref: 12177200 J Neurosci, 2002
- At the postsynaptic membrane of glutamatergic synapses, the cAMP dependent protein kinase  **PKA** , protein kinase C PKC, and calcineurin CaN anchoring protein AKAP79 150 is recruited to  **NMDA**  and AMPA glutamate receptors by postsynaptic density PSD 95 family membrane associated guanylate kinase MAGUK scaffold proteins.  Ref: 12177200 J Neurosci, 2002
- Here, we find that  **PKA**  and PKC phosphorylation sites flanking the RXR motif of the  **NMDA**  receptor NR1 subunit suppress ER retention and regulate receptor forward trafficking.  Ref: 14529714 Neuropharmacology, 2003
- Coordinated  **PKA**  and PKC phosphorylation suppresses RXR mediated ER retention and regulates the surface delivery of  **NMDA**  receptors.  Ref: 14529714 Neuropharmacology, 2003

**Parallel relationship** (e.g. studied together, co-existance, homology, etc.)

- The role of  **NMDA**  glutamate receptors,  **PKA** , MAPK, and CAMKII in the hippocampus in extinction of conditioned fear.  Ref: 12625457 Hippocampus, 2003
- The facilitation of hippocampus based, long lasting synaptic plasticity, which is frequently investigated in model systems such as long term potentiation LTP and in learning paradigms such as the Morris water maze, is associated with several cellular key events calcium influx through the N methyl D aspartate  **NMDA**  receptor, generation of cyclic AMP cAMP and activation of protein kinase A  **PKA** , phosphorylation of mitogen associated protein kinase MAPK and cAMP response element binding protein CREB, and subsequent transcription of plasticity associated genes.  Ref: 12668903 Mol Neurobiol, 2003
- Both tyrosine kinase and PKC... appear to be essential for EtOH withdrawal hyper responsiveness mediated by postsynaptic  **NMDA**  receptors in spinal cord motor neurons.  Ref: 12746225 Br J Pharmacol, 2003
- Regulation of DARPP 32 dephosphorylation at  **PKA**  and Cdk5 sites by  **NMDA**  and AMPA receptors distinct roles of calcineurin and protein phosphatase 2A.  Ref: 12065642 J Neurochem, 2002
- in the presence of H 89, a  **PKA**  inhibitor, the  **NMDA**  evoked calcium i increase was attenuated.  Ref: 12392174 Vis NeurosciVis Neurosci, 2002
- In particular, phosphorylation of GluR1, an AMPAR subunit, by  **PKA**  at serine 845 S845 increases peak open channel probability and is permissive for both the synaptic expression of the receptor and  **NMDA**  receptor NMDAR dependent long term potentiation LTP.  Ref: 12843287 J Neurosci, 2003
- These results indicate that ER retention of  **NMDA**  receptors is tightly regulated, and suggest that coordinated phosphorylation by  **PKA**  and PKC mediates release of receptors from the ER for subsequent traffic to synapses.  Ref: 14529714 Neuropharmacology, 2003
- Furthermore, it does not employ the adenylyl cyclase cAMP  **PKA**  cascade, the phospholipase C signalling pathway and  **NMDA**  receptor coupled mechanisms to depress excitatory synaptic transmission in the PBN.  Ref: 14672813 Brain Res, 2004
- **NMDA**  receptor activation also regulated dendritic spine localization of  **PKA**  and CaN and association of the AKAP79 150  **PKA**  complex with PSD MAGUKs.  Ref: 12177200 J Neurosci, 2002
- The new synapses are enriched in  **NMDA**  receptor and it was found that their generation involves activation of  **NMDA**  receptors,  **PKA**  and CREB.  Ref: 14560699 Neurol Neurochir Pol, 2003
